# Exploring the Gating Mechanism of the Human Copper Transporter, hCtr1, Using EPR Spectroscopy

**DOI:** 10.3390/biom15010127

**Published:** 2025-01-14

**Authors:** Shahaf Peleg, Shelly Meron, Yulia Shenberger, Lukas Hofmann, Lada Gevorkyan-Airapetov, Sharon Ruthstein

**Affiliations:** Department of Chemistry and Institute of Nanotechnology and Advanced Materials, Faculty of Exact Sciences, Bar-Ilan University, Ramat-Gan 5290002, Israel; shahaf.peleg@biu.ac.il (S.P.); shelly.meron@biu.ac.il (S.M.); yulia.shteinbok@biu.ac.il (Y.S.); lukas.hofmman@biu.ac.il (L.H.); gavorkl@biu.ac.il (L.G.-A.)

**Keywords:** copper transporter, copper metabolism, hCtr1, EPR spectroscopy

## Abstract

Ctr1 is a membrane-spanning homotrimer that facilitates copper uptake in eukaryotic cells with high affinity. While structural details of the transmembrane domain of human Ctr1 have been elucidated using X-ray crystallography and cryo-EM, the transfer mechanisms of copper and the conformational changes that control the gating mechanism remain poorly understood. The role of the extracellular N-terminal domains is particularly unclear due to the absence of a high-resolution structure of the full-length hCtr1 protein and limited biochemical and biophysical characterization of the transporter in solution and in cell. In this study, we employed distance electron paramagnetic resonance to investigate the conformational changes of the extracellular N-terminal domain of full-length hCtr1, both in vitro and in cells, as a function of Cu(I) binding. Our results demonstrate that at specific Cu(I) concentrations, the extracellular chains move closer to the lumen to facilitate copper transfer. Additionally, while at these concentrations the intracellular part is penetrating the lumen, suggesting a ball-and-chain gating mechanism. Moreover, this phenomenon was observed for both reconstituted protein in micelles and in native cell membranes. However, the measured distance values were slightly different, suggesting that the membrane’s characteristics and therefore its lipid composition also impact and even regulate the gating mechanism of hCtr1.

## 1. Introduction

The precise regulation of ion transport across biological membranes is crucial for all living cells. Ion channels and transporters are large transmembrane proteins that facilitate the selective movement of small inorganic ions. Resolving the mechanisms of the capturing, gating, and releasing of specific ions, which are governed by conformational changes within these proteins, is essential for understanding their cellular function [1,2,3].

The specificity of ion and ligand transporters is determined by the extracellular and intracellular domains involved in the transfer process. These domains often lack a defined secondary structure and are disordered, making it challenging to analyze the conformational changes they undergo during ligand or ion binding and transport. This dilemma impedes investigations of the gating mechanisms of these transporters. Electron paramagnetic resonance (EPR) spectroscopy has proven to be a powerful biophysical tool for obtaining high-resolution insights into these intricate biological systems. Recent work by the Cafiso and Pliotas groups has demonstrated that EPR spectroscopy can effectively track in situ conformational changes in the extracellular domains of membrane proteins within their native environment [4,5,6].

The human copper transporter, hCtr1, is the main gatekeeper of copper ions into the cells [7,8,9]. Copper is essential for cell survival; however, when its concentration is not tightly regulated, it can lead to toxicity and cell death. Therefore, the cellular systems hold restricted regulation systems, controlled by specific proteins that should shuttle the copper ions to specific subcellular locations. hCtr1 fulfills three distinct roles. In its first role, hCtr1 accumulates copper with an oxidation state of +2 from blood carrier proteins, such as human serum albumin [10,11,12]. In a reducing environment, the extracellular domain of hCtr1 facilitates the reduction of Cu(II) to Cu(I). In its last role, hCrt1 transports Cu(I) into the cell, where specific Cu(I) chaperones deliver it to the appropriate subcellular pathways [13,14,15,16]. The extracellular hCtr1 domain is characterized by His-rich sites [10,11,12], ^1^MDHSHH and ^22^HHH segments, and Met-based motifs, ^7^MGMSYM and ^41^MMMPM [17,18,19], that coordinate copper in an oxidation state of Cu(II) and Cu(I), respectively. Cryo-EM and X-ray crystallography have provided structural information of the hCtr1 transmembrane domain [15,20]. However, the extracellular and intracellular domains are missing from these structures. Recently, all-atom molecular dynamics (MD) simulations suggested that the extracellular domain is disordered and, upon Cu(I) binding, the extracellular domain approaches the selectivity filter, which leads to the opening of the transporter and conformational changes in the transmembrane helices [21].

We recently showed that each monomer of the hCtr1 extracellular domains binds two Cu(II) ions, resulting in a total of six Cu(II) ions per hCtr1 trimer. These results were derived from various in vitro EPR, UV-Vis measurements, and MD simulations on the full-length hCtr1 protein [22]. We also showed that a hCtr1 monomer can coordinate up to five Cu(I) ions and that the intracellular domain of hCtr1 occupies various conformational states as a function of Cu(I) concentration. More specifically, the intracellular domain is highly dynamic in its apo-state; however, at a ratio of 2–3 Cu(I):hCtr1 monomer, the intracellular C-terminal tail is folded inside the hCtr1 lumen and a homogeneous rigid structure is obtained. Subsequently, the C-terminal is released from the hCtr1 lumen upon increasing the Cu(I) concentration.

**Figure 1:**
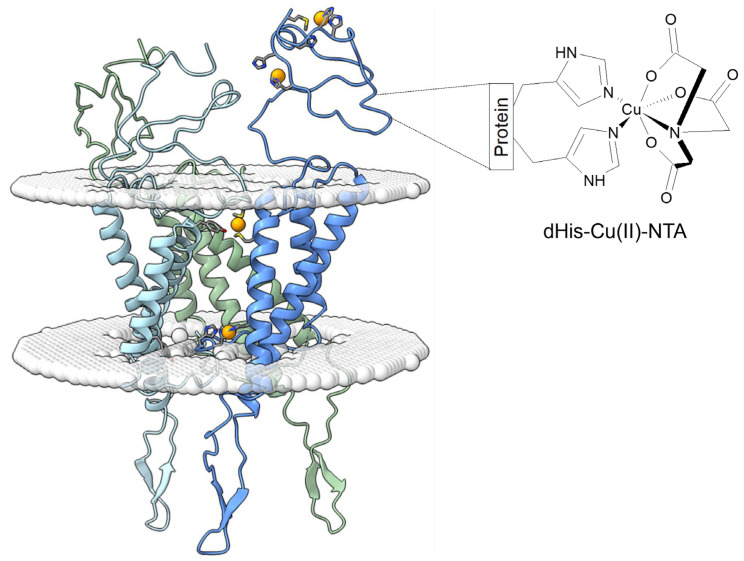
Schematic drawing of hCtr1 trimer. The orange balls represent Cu(I) ions. The zoom on the right-hand side illustrates the used histidine residues in complex with Cu(II)-NTA spin labeling used as spin label. The trimeric hCtr1 model is based on the structure of Ctr1 from *Salmo salar* PDB-ID: 6M98 [20]. The model was generated with Swiss-Model and prepared with Pymol (Version 2.6, Schrödinger, LLC., New York, NY, USA) and UCSF ChimeraX (Version 1.9, UCSF, CA, USA) [31].

This study aims to experimentally target conformational changes in the extracellular domain of the full-length hCtr1 protein in vitro and in situ using distance EPR measurements. Paramagnetic sites were added to follow the conformational changes of the hCtr1 extracellular domain using EPR measurements. Cu(II) is a paramagnetic metal ion and in general its binding to the extracellular domain of hCtr1 can be used to gain structural information on the extracellular domain. However, since these measurements are performed in the presence of Cu(I) ions, there might be a redox reaction between them; moreover, Cu(II) is unstable in the reducing environment of the cell and is readily reduced to Cu(I) [23,24]. In order to ensure that Cu(II) bound to the extracellular domain and was not reduced, Cu(II) was protected with a nitrilamino acid (NTA) ligand (Figure 1). The group of Saxena was the first to introduce this methodology for EPR measurements [25,26] and the group of Bode has shown the high affinity of Cu(II)-NTA to His-rich sites [27,28,29]. The advantage of such a spin labeling methodology is that it is positioned on the backbone of the protein, with little flexibility, which leads to a four- to five-times narrower distance distribution function as compared to the common nitroxide spin labels [30]. In a recent manuscript, we illustrated how Cu(II)-NTA and dHis sites can be employed to detect conformational changes in proteins within the cellular environment [24]. Furthermore, previously we showed that Cu(II)-NTA binds to hCtr1 with a coordination site similar to that of free Cu(II) and involves at least one histidine residue from the extracellular domain of hCtr1 [3,24]. Moreover, these two recent publications demonstrated an almost complete absence of non-specific Cu(II)-NTA binding.

Here, we utilized the Cu(II)-NTA spin-labeling technique coupled with distance EPR measurements to observe conformational changes in the extracellular domain of hCtr1. This was accomplished for both in vitro with protein reconstituted in micelles and in situ with overexpressed protein in *Sf9* insect cells. This methodology was employed to investigate the gating mechanism of hCtr1 in response to varying Cu(I) concentrations. The results highlighted the sensitivity of EPR spectroscopy in tracking conformational changes in disordered domains, offering valuable insights into the selectivity and transport mechanisms of ion-gated channels and transporters.

## 2. Materials and Methods

### 2.1. Cloning, Expression, and Purification of hCtr1 for In Vitro Experiments

The expression of hCtr1 was described in detail in our previous publications [3,22]. In short, wild-type (WT) hCtr1 was generated by PCR amplification and inserted into a modified pFastBac (pK503-9) vector with an N-terminal FLAG tag. To produce baculovirus for hCtr1 expression, recombinant bacmid DNA was extracted and transfected into *Sf9* insect cells (Expression Systems, LLC, Davis, CA, USA) using Cellfectin II Reagent (Thermo Fisher, Airport City, IL, USA), following the Bac-to-Bac instruction manual (Thermo Fisher, Airport City, IL, USA). The cells were cultured at 27 °C for three days and then harvested and re-suspended in a buffer containing 400 mM NaCl, 10% glycerol, and 20 mM HEPES (Sigma-Aldrich, Rehovot, IL, USA) at pH 7.4. After lysis, the resulting pellet was re-suspended in a buffer containing 1.5% Triton X-100, 200 mM NaCl, 10% glycerol, and 20 mM HEPES (pH 7.4) and incubated overnight at 4 °C. After incubation, the suspension was centrifuged again at 40,000 rpm for 40 min. The supernatant was supplemented with 3 mM CaCl_2_ and loaded onto an anti-FLAG M1 agarose affinity gel column (Sigma-Aldrich, Rehovot, IL, USA) overnight at 4 °C, pre-equilibrated with TBS buffer (150 mM NaCl, 50 mM Tris-HCl, pH 7.4). Finally, the column was washed with TBS buffer and eluted with a buffer containing 5 mM EDTA.

To prepare the Cu(II)-NTA solution, 10 mM Cu(II) was combined with 10 mM NTA and mixed overnight. Subsequently, 240 µM Cu(II)-NTA was added to 120 µM purified hCtr1 solution and incubated overnight at 4 °C.

The addition of Cu(I) was performed as follows: concentrated Cu(I) solution (30 mM) was first prepared using Tetrakis(acetonitrile)copper(I) hexafluorophosphate (Sigma-Aldrich, Rehovot, IL, USA) dissolved in dry acetonitrile (HPLC grade) under anaerobic conditions. The Cu(I) solution was then added at different volumes to 120 µM hCtr1 monomer to give 1:1 Cu(I):hCtr1, 3:1 Cu(I):hCtr1, 5:1 Cu(I):hCtr1 ratios. Twenty percent glycerol was added to all samples.

### 2.2. hCtr1 In Situ and Cell Membrane Fragment Experiments

For in situ measurements according to the protocol described in [3], after the incubation of *Sf9* insect cells and hCtr1 expression, the cells were centrifuged at 1000 rpm for 5 min at 21 °C and the resulting pellet was resuspended in 30 mL of insect cell medium for a final concentration of 28.3 × 10^6^ cells/mL. The suspension was then divided into ten test tubes, each containing 5 mL of medium with intact cells. A 250 µM Cu(II)-NTA solution was added to each tube and the samples were incubated overnight at room temperature with shaking. Following incubation, the cells were washed twice with fresh medium and subsequently divided into four samples for further analysis. Aliquots of 30 mM Cu(I) solution were then added into two samples at concentrations of 360 µM and 600 µM. A sample from each concentration was incubated for half an hour and two hours respectively. Twenty percent glycerol was added to all samples. Additional samples were lysed and subjected to ultracentrifugation at 40,000 rpm and 4 °C for 45 min.

### 2.3. Q-Band Double Electron–Electron Resonance (DEER) Experiments

DEER experiments (π/2(ν_obs_) − τ1 − π(ν_obs_) − t′ − π(ν_pump_) − (τ1 + τ2 − t′) − π(ν_obs_) − τ2 − echo) were carried out with rectangular pulses without an AWG system at 20 ± 1.0 K on a Q-band Elexsys E580 spectrometer (equipped with a 2 mm probe head). A two-step phase cycle was employed on the first pulse. The echo was measured as a function of t′, whereas τ1 was set to 200 ns. τ_2_ was between 2500–4000 ns and was chosen based on the echo intensity and relaxation time; the exact value of τ_2_ was taken into consideration in the distance distribution error analysis, as implemented in DeerAnalysis. The durations of the observer π/2 and π pulses were 14 and 28 ns, respectively, while the π pump pulse was also 28 ns. The observer frequency was constant and set to 33.85 GHz for all experiments, while the pump frequency was set to 33.74 GHz and the magnetic field was 11,680 G. Previous studies have shown that within the range of 500 G, orientational effects can be ignored at the g_^_ region [32,33]. The data were analyzed using the DeerAnalysis 2019 program (ETH, Switzerland). Tikhonov regularization and DeerNet were also used to analyze the data [34]. The x-axis in the distance distribution was corrected based on the g-value and was multiplied by (g_nitroxide_/g_⊥,Cu_)^0.67^ = 0.986; where g_nitroxide_ = 2.0057, and g_⊥,Cu_ = 2.05 [32].

## 3. Results

The full-length hCtr1 was expressed in insect cells and Cu(II)-NTA was added either to cells (expressing hCtr1) or to purified protein at a ratio of 2:1 Cu(II)-NTA:hCtr1, as was also described in our previous publication [3]. Cu(I) was then added to the cells or hCtr1 monomer at different ratios as described in the materials and methods section under anaerobic conditions. An SDS-Gel picture of the purified hCtr1 is shown in Figure S1, Supplementary Materials. Western blot experiments were carried out to ensure that the presence of Cu(II)-NTA and Cu(I) does not affect hCtr1 expression (Figure S2, Supplementary Materials).

**Figure 2:**
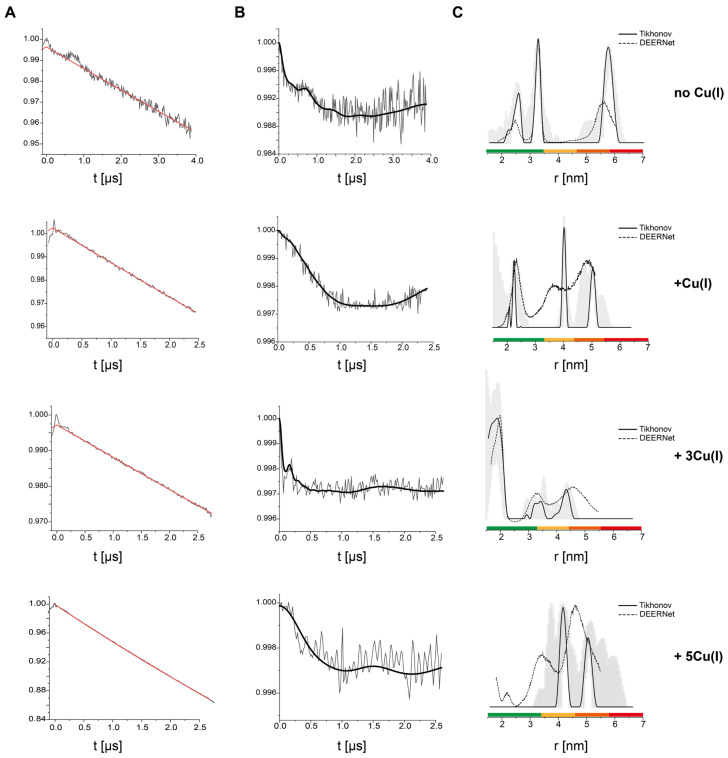
Q-band DEER measurements on purified hCtr1 upon Cu(I) coordination. (**A**) Raw time domain DEER data (black) and the background function (red). (**B**) DEER time domain data after background correction and the corresponding fit. (**C**) The corresponding distance distributions. The data were analyzed using the DeerAnalysis program using Tikhonov regularization with a regularization parameter of 20 (solid black lines) and DEERNet (dashed black lines). Distance distribution validation considered white noise, background start. and dimensionality. The differences between the Tikhonov analysis and DEERNet are within the margin of error. The color bar indicates reliability ranges (green: shape reliable; yellow: mean and width reliable; orange: mean reliable; red: no quantification possible). The data were acquired at 20 K, where 2:1 Cu(II)-NTA to 120 µM hCtr1 monomer in HEPES buffer, pH 7.4, and 20% glycerol were added. Cu(I) was added at different ratios as compared to hCtr1 monomer. The data for the apo hCtr1 (no Cu(I)) were reproduced from Meron et al. 2024 with permission from ACS [3].

Double electron–electron resonance (DEER) pulsed EPR distance measurements were conducted to evaluate the distance distributions between Cu(II)-NTA sites of the hCtr1 extracellular domains. Initially, the DEER measurements were performed on purified hCtr1 reconstituted in triton micelles (Figure 2) as a function of [Cu(I)]. The DEER data suggest for the apo hCtr1 (no Cu(I) added) a bimodal distribution between 2.0–3.5 nm and an additional distribution around a longer distance of 5.6 nm. The addition of small amounts of Cu(I) in a ratio of 1:1 Cu(I):hCtr1 monomer results in distance distributions around 2.1 nm and between 4.0–5.0 nm. However, the addition of larger amounts of Cu(I) to the solution in a ratio of 3Cu(I):hCtr1 monomer results in a single distance distribution function around 1.8 nm (repeated experiments for this concentration are provided in Figure S3, Supplementary Materials). The addition of 5Cu(I):hCtr1 monomer reveals only the distribution at longer distances between 4.0–5.0 nm. This phenomenon is very similar to what was detected for the intracellular domain of hCtr1 at a 3Cu(I):hCtr1 ratio. While at other copper concentrations lower or higher than 3Cu(I):hCtr1 monomer, various distance distribution functions appear at longer distances [22]. In this study, MD simulations suggested that such homogeneous small-distance distribution can only occur when the three intracellular tails of hCtr1 enter the lumen to transfer Cu(I) from the lumen to the cell [22]. Recently, MD simulations on the extracellular domain of hCtr1 also suggested that the extracellular domain must approach the hCtr1 funnel to allow for Cu(I) ion transfer [21]. Altogether, the EPR and MD data support the notion that at a ratio of 3Cu(I):hCtr1, both the extracellular and the intracellular domains should approach the hCtr1 lumen to allow fast copper transfer into the cell.

**Figure 3:**
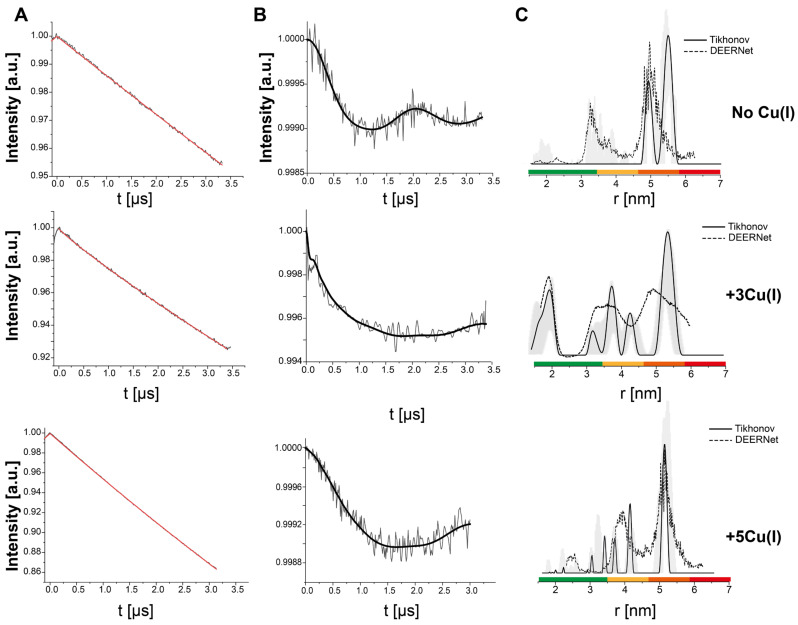
Q-band DEER measurements on Cu(II)-NTA in situ of hCtr1 upon Cu(I) coordination. (**A**) Raw time domain DEER data (black) and the background function (red). (**B**) DEER time domain data after background correction and the corresponding fit. (**C**) The corresponding distance distributions. The data were analyzed using the DeerAnalysis program using Tikhonov regularization with a regularization parameter of 20 (solid black lines) and DEERNet (dashed black lines). Distance distribution validation considered white noise, background start, and dimensionality. The differences between the Tikhonov analysis and DEERNet are within the margin of error. The color bar indicates reliability ranges (green: shape reliable; yellow: mean and width reliable; orange: mean reliable; red: no quantification possible). The data were acquired at 20 K, where 240 μM Cu(II)-NTA was added to the cells, 20% glycerol, and different Cu(I) concentrations (360 μM/600 μM). The data for the apo hCtr1 (no Cu(I)) were reproduced from Meron et al. 2024 with permission from ACS [3].

Next, for in situ measurements, Q-band DEER experiments were performed with Cu(II)-NTA bound to hCtr1 in the cells. To begin, 240 µM Cu(II)-NTA was added to the cells (in our expression protocol, the amount of purified hCtr1 was in the range between 120–140 µM) and Cu(I) was added to the cells at different concentrations (360/600 µM, corresponding to 3Cu(I):hCtr1 monomer or 5Cu(I):hCtr1 monomer). The DEER data for the cells are presented in Figure 3. In the absence of Cu(I), the DEER data suggest distributions of around 4.8–6.0 nm. The smaller distances that were observed for the purified protein in micelles between 2.0–3.5 nm were not detected in the cells. As previously discussed [3], we conclude that in the native environment, the extracellular chains are in proximity to the functional group of the phospholipids and associated with the membrane, resulting in a more distant organization of the three disordered N-termini. Conversely, once the protein is reconstituted in micelles, this anchoring is disrupted, which led to the observed close configuration of the three extracellular domains of hCtr1.

DEER measurements were next performed in the presence of Cu(I). The addition of copper at a ratio of 3Cu(I):hCtr1 suggested a distribution of around 1.8 nm, similar to the purified protein at this concentration (repeated experiments for this concentration are provided in Figure S4, Supplementary Materials), while at higher amounts of copper, this distribution disappears and distributions between 3.8–5.5 nm appear. Although there are some differences in the precise values of the distances between the in vitro and in situ EPR measurements, the observation that the extracellular chains are approaching each other at the same specific concentration in both systems is striking. The changes in the distance values highlight the importance of the lipids or surfactants in the function of gating transporters [35,36].

**Figure 4:**
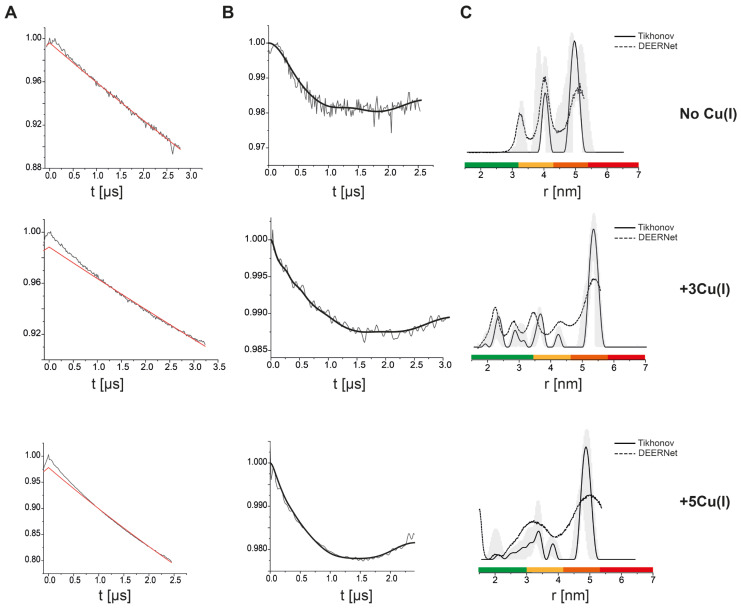
Q-band DEER measurements on Cu(II)-NTA bound to hCtr1 in cell membrane fragments as a function of Cu(I) coordination. (**A**) Raw time domain DEER data (black) and the background function (red). (**B**) DEER time domain data after background correction and the corresponding fit. (**C**) The corresponding distance distributions. The data were analyzed using the DeerAnalysis program using Tikhonov regularization with a regularization parameter of 20 (solid black lines) and DEERNet (dashed black lines). Distance distribution validation considered white noise, background start, and dimensionality. The differences between the Tikhonov analysis and DEERNet are within the margin of error. The color bar indicates reliability ranges (green: shape reliable; yellow: mean and width reliable; orange: mean reliable; red: no quantification possible). The data were acquired at 20 K, where 240 μM Cu(II)-NTA, 20% glycerol, and different Cu(I) concentrations (360 μM/600 μM) were added to the cells and the cells were then lysed and centrifuged. The data for the apo hCtr1 (no Cu(I)) were reproduced from Meron et al. 2024 with permission from ACS [3].

In order to further verify this, we lysed the cells after adding Cu(II)-NTA and Cu(I) and EPR measurements were conducted (Figure 4). Without Cu(I), the DEER data suggest a bimodal distance distribution between 4.0–5.5 nm, similar to the in situ cells experiment. The addition of 3Cu(I):hCtr1 resulted in additional distributions with smaller distances between 2.0–4.0 nm. Adding 5Cu(I):hCtr1 led to a reduction in the contributions of the distributions around 2.0 nm, which agrees well with the findings on the purified and in situ hCtr1 experiments. Pulsed EPR measurements were also conducted on *sf9* cells without hCtr1 overexpression (Figure S5, Supplementary Materials). In this case, the echo intensity was five times lower, as the Cu(II)-NTA was washed away, confirming that there is no specific binding without hCtr1; moreover, no signal at all was detected in the cell membrane fragments. The relaxation time of the non-bound Cu(II)-NTA in the cells is much faster and its contribution to the DEER signal is negligible (Figure S5, Supplementary Materials).

The modulation depth detected here, which is related to the excitation profile of the paramagnetic centers, is very low. In general, Cu(II) distance measurements are characterized by a low modulation depth, owing to the large spectral width of Cu(II) [37]. Here, the limitation is even larger owing to the fact that the studied system is a human membrane transporter with a comparable low expression yield [3]. Despite this limitation, the use of this spin-labeling methodology allows for comparable highly resolved DEER data, due to the orthogonal labeling, which reduces the flexibility of the spin labeling and allows narrow distance distribution functions [24,33,37]. In the purified protein, the modulation depth is ~1–2%, which is larger than that in the cellular environment (~0.1%). This suggests that less Cu(II)-NTA molecules are bound to the extracellular domain of the hCtr1 trimer in the cellular environment compared to the reconstituted hCtr1 in micelles.

**Figure 5:**
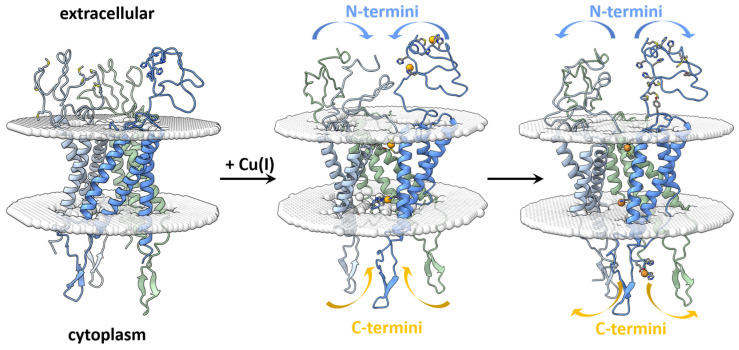
The copper transfer mechanism through hCtr1 transporter. Upon Cu(I) uptake, the extracellular chains move closer to each other and the intracellular chains move into the lumen (closed state). Once Cu(I) is transferred into the lumen, the extracellular chains are spread apart, with the intracellular chains pointing into the cytoplasmic domain for the transfer of copper to the various metallochaperones (opened state). This figure was created as described in Figure 1 and prepared using UCSF ChimeraX.

Despite the experimental limitations, DEER experiments were able to follow conformational changes in vitro and in situ of the disordered domains of extracellular hCtr1 upon Cu(I) binding. Figure 5 illustrates the proposed copper gating mechanism of copper through the hCtr1 transporter. In the apo-state, the extracellular N-termini chains are further apart and the intracellular chains are in the cytoplasmic domain (opened state).

At specific Cu(I) concentrations, the extracellular chains move closer to each other and the intracellular chains move toward the protein lumen (closed state). At higher copper concentrations, the intracellular chains are released back to the cytoplasmic side and the extracellular domains move further apart, enabling them to scavenge more copper ions.

## 4. Discussion

Gaining structural information on ion transporters within the cell is a complex task, particularly when focusing on the extracellular domains that regulate specificity and gating mechanisms. These regions often lack a well-defined secondary structure and are intrinsically disordered, making it challenging to study their conformational changes. The difficulty is increased when the experiments are performed in the cellular environment and are compared to the micellar environment. In micelles, proteins may adopt conformations or interact differently compared to their behavior in the cell, potentially leading to discrepancies in the observed gating mechanisms. In contrast, studying transporters within the cell provides a more physiologically relevant setting but introduces additional variables and complexities, such as interactions with other cellular components and the dynamic nature of the cellular environment [35,36,38]. Thus, accurately capturing the structural dynamics of extracellular domains and their role in specificity and gating mechanisms requires careful consideration of these experimental contexts and the limitations they impose.

EPR spectroscopy is a powerful biophysical tool for studying conformational changes in complex biological systems and transmembrane systems that are difficult to monitor with other methods. EPR is particularly useful for tracking conformational changes in disordered domains of transporters and channels [4,5,6,22] or following a transcription mechanism [32,39]. We recently demonstrated the use of Cu(II)-NTA and dHis sites as spin-labeling methodologies for EPR distance measurements to investigate conformational changes of proteins in cellular conditions [3,24]. Here, we applied this approach to monitor structural changes in the extracellular domain of the human copper transporter, hCtr1. The Cu(II)-NTA spin-labeling method provides several advantages for studying hCtr1 in its cellular environment: first, multiple native histidine residues in the extracellular domains of hCtr1 serve as binding sites. Second, EPR data indicate that Cu(II)-NTA binds to hCtr1 similarly as to free Cu(II). Because NTA protects Cu(II) from reduction and from reacting with free Cu(I) ions, this spin-labeling approach allows us to track conformational changes in hCtr1 in response to Cu(I) binding. Our EPR distance measurements indicate that the extracellular chains of hCtr1 come closer together upon Cu(I) binding, both in purified hCtr1 within micelles and in the native cell membrane. Notably, at a specific ratio of three Cu(I) ions to one hCtr1 monomer, the extracellular chains exhibit a single distance distribution function. This finding suggests that all chains simultaneously move closer to the hCtr1 lumen. Previously, similar findings proposed that at this specific Cu(I) concentration, the intracellular C-terminal chains also approach the hCtr1 lumen to facilitate copper ion transfer [22].

The gating mechanisms of ion-gated transporters such as potassium and calcium channels have been extensively studied using various biophysical and computational methods. These studies indicate that gating is regulated by a synergy between the high affinity of the extracellular domain for specific ions, dynamic and conformational changes in the extracellular domain that lead to interactions with the membrane, and alterations in the orientation of transmembrane helices and the penetration of the intracellular loop into the lumen, a mechanism often referred to as the “ball-and-chain” mechanism [40,41]. This mechanism is analogous to our observations of copper gating by hCtr1. We observed transitions between open and closed states in both the extracellular and intracellular regions at specific copper concentrations. In the closed state, characterized by close interactions among all extracellular domains and the penetration of the intracellular domain into the lumen, we also noted that the membrane environment influenced these interactions. Specifically, EPR measurements in Triton micelles revealed closer interactions among the extracellular chains compared to those observed in the native cell membrane.

## 5. Conclusions

EPR measurements conducted on the full-length human copper transporter, hCtr1, in reconstituted protein in micelles and expressed protein in insect cells as a function of Cu(I) ion concentration suggested a gating mechanism involving opened and closed states, where in the closed state the extracellular chains approach each other and the intracellular chain is penetrating the lumen. These two states were present for the reconstituted protein in triton micelles and in the native membrane. However, the distance distribution values were slightly different between these two states, also highlighting the importance of the membrane characteristic to the gating mechanism.

## Data Availability

The original contributions presented in this study are included in the article/Supplementary Materials. Further inquiries can be directed to the corresponding author.

## Supplementary Materials

The following supporting information can be downloaded at: https://www.mdpi.com/article/10.3390/biom15010127/s1, Figure S1: SDS-PAGE analysis of purified WT-hCtr1 following silver staining. Lanes 2–6 display the elution fractions obtained from an anti-FLAG M1 agarose affinity gel column using a solution containing 5 mM EDTA and 100 μg/mL FLAG peptide; Figure S2: Western blot of WT-hCtr1 with Cu(II)-NTA complex and Cu(I)-tetrakis at different ratios; Figure S3: Various DEER time domain signals and corresponding distance distributions functions were acquired on different samples of purified hCtr1 in the presence of Cu(II)-NTA and 3Cu(I); Figure S4: Various DEER time domain signals and corresponding distance distributions functions were acquired on different samples of overexpressed hCtr1 in the cells, in the presence of Cu(II)-NTA and 3Cu(I); Figure S5: (A). Two-pulse echo decay for Cu(II)-NTA in sf9 cells with (red) and without hCtr1 expression (black). (B). DEER time domain signal for Cu(II)-NTA in sf9 cells with (red) and without hCtr1 expression (black); Figure S6. Original Western Blot image ctr1 in insect cells.
