# Supplementary material for: Exploring the Gating Mechanism of the Human Copper Transporter, hCtr1, Using EPR Spectroscopy
Source: Biomolecules. 2025 Jan 14;15(1):127. doi: 10.3390/biom15010127 (PMC11763644; doi:10.3390/biom15010127)
Supplement: Supplementary file 1 [file biomolecules-15-00127-s001.zip › biomolecules-3338651-supplementary.pdf]

SUPPLEMENTARY MATERIALS

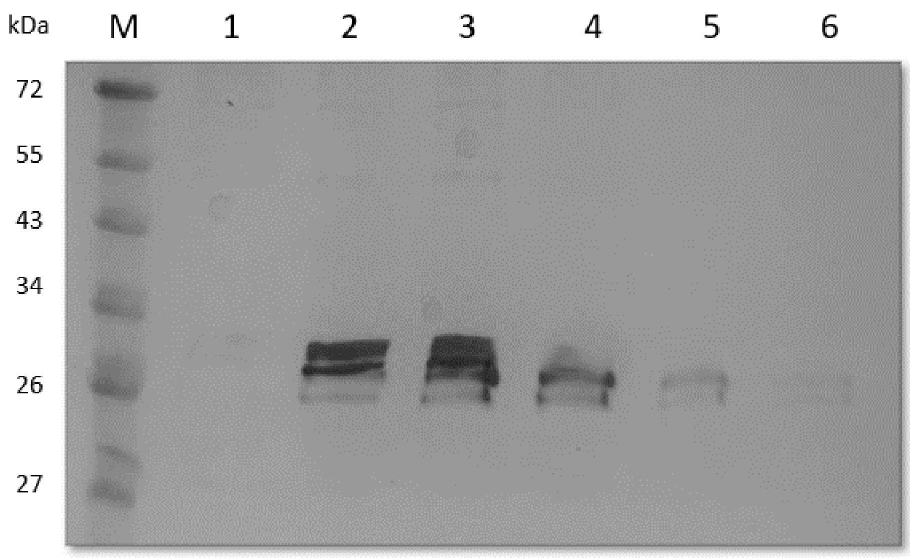

Figure S1. SDS-PAGE analysis of purified WT-hCtr1 following silver staining. Lanes 2–6 display the elution fractions obtained from an anti-FLAG M1 agarose affinity gel column using a solution containing 5 mM EDTA and 100 μg/mL FLAG peptide.

Western blot:

Western blot experiments were carried out according to protocol published earlier [1] for overexpressed hCtr1 protein in insect cells in the presence of Cu(II)-NTA complex and Cu(I)-tetrakis (Sigma-Aldrich) to ensure that the presence of Cu(II)-NTA and Cu(I) still express hCtr1 protein.

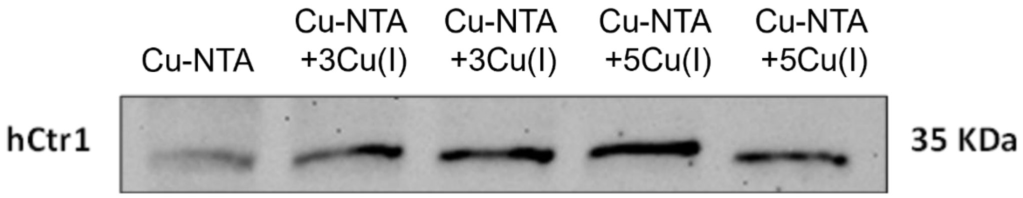

Figure S2. Western blot of WT-hCtr1 with Cu(II)-NTA complex and Cu(I)-tetrakis at different ratios.

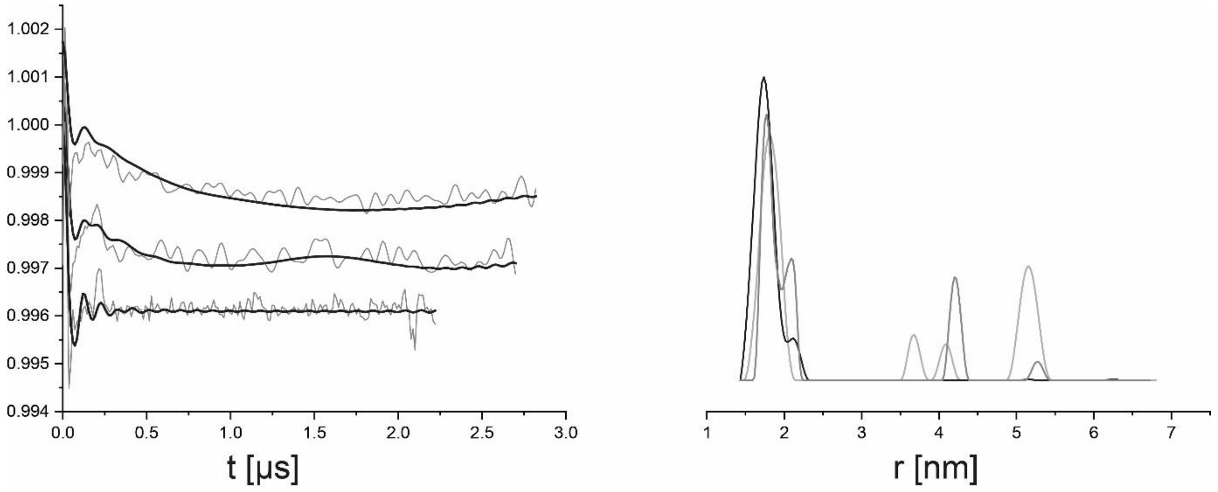

Figure S3. Various DEER time domain signals and corresponding distance distributions functions were acquired on different samples of purified hCtr1 in the presence of Cu(II)-NTA and 3Cu(I).

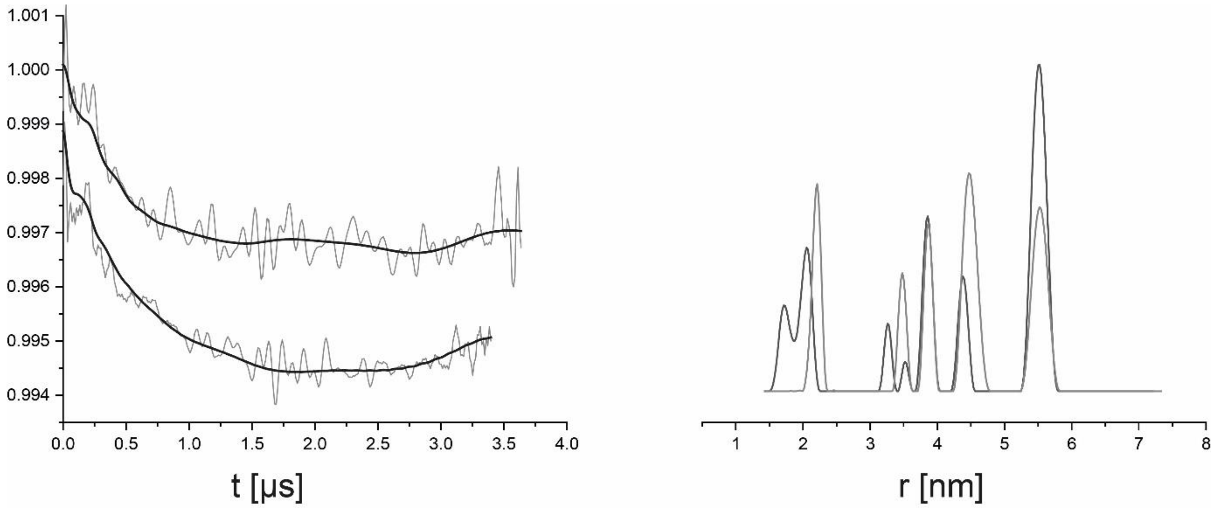

Figure S4. Various DEER time domain signals and corresponding distance distributions functions were acquired on different samples of overexpressed hCtr1 in the cells, in the presence of Cu(II)-NTA and 3Cu(I).

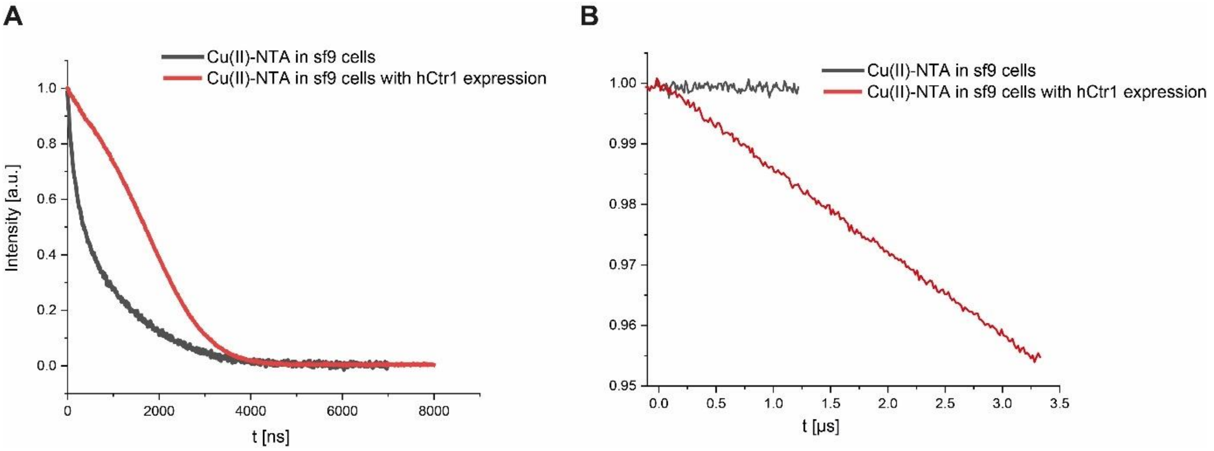

Figure S5. A. Two-pulse echo decay for Cu(II)-NTA in sf9 cells with (red) and without hCtr1 expression (black). **B**. DEER time domain signal for Cu(II)-NTA in sf9 cells with (red) and without hCtr1 expression (black).

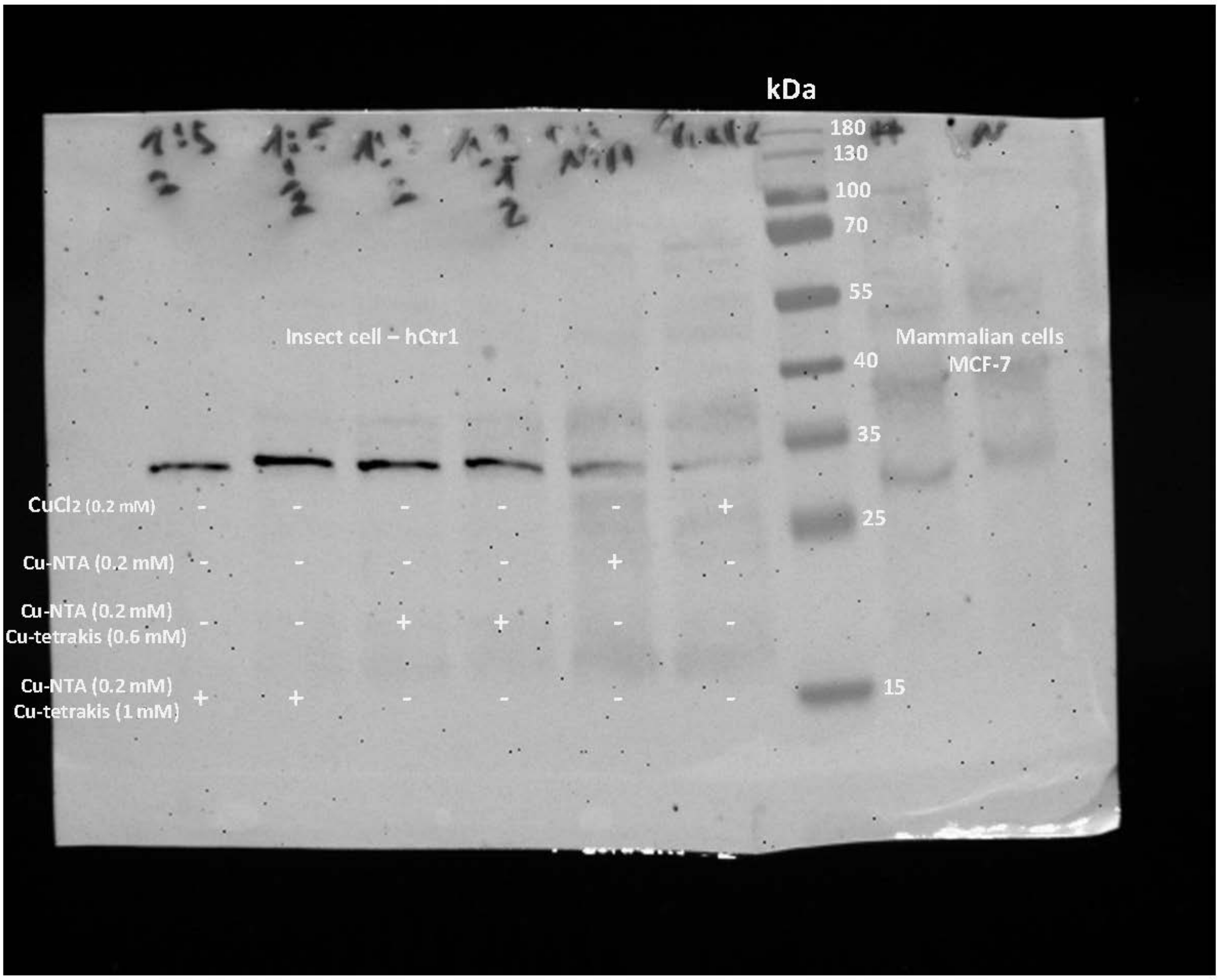

Figure S6. Original Western Blot image ctr1 in insect cells.

Reference

1. Meron, S.; Peleg, S.; Shenberger, Y.; Hofmann, L.; Gevorkyan-Airapetov, L.; Ruthstein, S. Tracking Disordered Extracellular Domains of Membrane Proteins in the Cell with Cu(II)-Based Spin Labels. J Phys Chem B 2024, 128, 8908-8914, doi:10.1021/acs.jpcb.4c03676.
